# Estimating mean circulatory filling pressure in humans: Physiological constraints on equilibrium‐based methods

**DOI:** 10.14814/phy2.70824

**Published:** 2026-03-16

**Authors:** L. M. van Loon, D. W. Donker, J. Lemson

**Affiliations:** ^1^ Cardiovascular and Respiratory Physiology, Technical Medical Centre University of Twente Enschede The Netherlands; ^2^ Department of Intensive Care Medicine Radboud University Medical Center Nijmegen The Netherlands; ^3^ Intensive Care Center University Medical Center Utrecht Utrecht The Netherlands

We thank Möller, Åneman, and Søndergaard for their thoughtful Letter to the Editor (Möller Per Werner, 2026). We agree with their central methodological point: an equilibrium filling pressure in the classical sense is only defined after rapid redistribution has abolished arteriovenous pressure gradients at zero flow, and it must be obtained within seconds—before baroreflex activation and evolving ischemia materially change vascular resistance, capacitance, and stressed volume (Möller Per Werner, 2026).

Our motivation is that much contemporary experimental and clinical work cites the equilibrium definition while leaving implicit the conditions required to realize it. Rothe's formulation is explicit: mean circulatory filling pressure is defined only after cessation of cardiac output and redistribution such that pressures are uniform throughout the system (Rothe, 1985). Guyton's insistence on measurement “immediately” after pump cessation was therefore not stylistic; it was necessary to interrogate the pre‐arrest volume–capacitance state rather than the rapidly evolving post‐arrest physiology (Guyton et al., 1954). Möller et al. correctly note, Guyton enforced early equilibration with immediate arteriovenous shunting. This is not a minor technical detail—it is a large perturbation that actively creates the single‐pressure condition assumed by the definition.

Accordingly, “equilibration” without forced redistribution is neither guaranteed nor passive on the relevant timescale. Baroreceptor transduction is beat‐to‐beat, with vagal modulation within the next cardiac cycle, and sympathetic vasomotor adjustments evolve within seconds (≈10 s for vascular resistance in controls) (Karemaker & DeBoer, 2017). Thus, unless equalization is forced extremely rapidly, the determinants of any equilibrium filling pressure—tone‐dependent compliance/capacitance and hence stressed volume—are already time‐varying during the interval used to infer “equilibrium” (Karemaker & DeBoer, 2017).

Human evidence supports this. Without shunting, post‐arrest pressures do not reliably converge to equilibrium within feasible windows. During induced ventricular fibrillation in 82 anesthetized patients, Schipke et al. observed persistent arterial–venous disequilibrium throughout 13–20 s and concluded that true equilibration would require longer no‐flow durations than are ethically possible in humans (Schipke et al., 2008). In ICU patients, Repessé et al. reported wide dispersion in systemic filling pressures at 1 min after death, with higher values after norepinephrine exposure, indicating strong tone dependence rather than a unique equilibrium constant (Repesse et al., 2015).

Against this background, we endorse the caution that pressures recorded without rapid shunting and complete equilibration must not be mistaken for mean circulatory filling pressure (MCFP). Where we diverge is the implication that omitting shunting yields merely a “suboptimal” estimate of the same equilibrium quantity. Our data show a qualitative incompatibility: after pump failure, venous pressure can exceed arterial pressure (a sustained retrograde gradient). This is not delayed convergence; it indicates functional partitioning, with pressure communication constrained by state‐dependent choke points. In that regime, the premise of a single‐pressure equilibrium description is violated.

This is consistent with established vascular mechanics. Collapsible vessels can exhibit waterfall (critical closing) behavior such that upstream and downstream pressures decouple and persistent gradients exist even at zero flow. Magder further demonstrated that Starling‐resistor dynamics can produce non‐intuitive zero‐flow pressure relations that reflect mechanical constraints rather than uniform equilibration (Magder, 1990). In parallel, Brengelmann cautioned against treating Guyton‐style venous return curves as evidence for a literal upstream reservoir pressure persisting during flow (Brengelmann, 2003). The implication is direct: if post‐arrest pressures enter a partitioned, waterfall‐dominated regime, then an arteriovenous shunt does not reveal a naturally emergent equilibrium of the intact circulation; it enforces one by bypassing the nonlinearities that govern pressure communication in vivo and may obscure the upstream venous pressure relevant for venous return.

We do not dispute that model‐based surrogates (including Pmsa‐type approaches) can be internally precise and clinically useful within their assumptions (Maas et al., 2012; van Loon et al., 2020). Our point is narrower: without enforced rapid equilibration, post‐arrest arterial and venous pressures need not be noisy equilibrium estimates; they can represent a different physiological regime shaped by reflexes and compartmentalized mechanics evolving on the same timescale. This is where computational physiology modeling (CPM) is particularly valuable: it enables mechanistic attribution of time‐resolved trajectories and tests whether observed dynamics require active regulation rather than passive compliance discharge, thereby distinguishing non‐equivalence from mere imprecision.

In summary, we agree on the definition and on the need for rapid shunting if the goal is to measure an equilibrium filling pressure. Our contribution is to demonstrate—via persistent arteriovenous disequilibrium and even pressure‐gradient reversal—that when this condition is absent (as is common and often unavoidable in humans), post‐arrest pressures predominantly reflect evolving reflexes and compartmentalized vascular mechanics rather than an equilibrium proxy for the pre‐arrest volume state.

## AUTHOR CONTRIBUTIONS

L.M. van Loon wrote the first draft. All authors revised and approved the manuscript.

## FUNDING INFORMATION

The authors have nothing to report.

## CONFLICT OF INTEREST STATEMENT

The authors declare no conflicts of interest.
